# 3-Formylchromone Counteracts STAT3 Signaling Pathway by Elevating SHP-2 Expression in Hepatocellular Carcinoma

**DOI:** 10.3390/biology11010029

**Published:** 2021-12-26

**Authors:** Chakrabhavi Dhananjaya Mohan, Min Hee Yang, Shobith Rangappa, Arunachalam Chinnathambi, Sulaiman Ali Alharbi, Tahani Awad Alahmadi, Amudha Deivasigamani, Kam Man Hui, Gautam Sethi, Kanchugarakoppal S. Rangappa, Kwang Seok Ahn

**Affiliations:** 1 Department of Studies in Molecular Biology, University of Mysore, Manasagangotri, Mysore 570006, India; mohan@biochemistry.uni-mysore.ac.in; 2 KHU-KIST Department of Converging Science and Technology, Kyung Hee University, Seoul 02447, Korea; didmini@naver.com; 3 Department of Science in Korean Medicine, Kyung Hee University, Seoul 02447, Korea; 4 Adichunchanagiri Institute for Molecular Medicine, Adichunchanagiri University, BG Nagara 571448, India; aimm@acu.edu.in; 5 Department of Botany and Microbiology, College of Science, King Saud University, Riyadh 11451, Saudi Arabia; carunachalam@ksu.edu.sa (A.C.); sharbi@ksu.edu.sa (S.A.A.); 6 Department of Pediatrics, College of Medicine and King Khalid University Hospital, King Saud University, Riyadh 11461, Saudi Arabia; talahmadi@ksu.edu.sa; 7 Division of Cellular and Molecular Research, Humphrey Oei Institute of Cancer Research, National Cancer Centre, Singapore 169610, Singapore; amudha.deivasigamani@nccs.com.sg (A.D.); cmrhkm@nccs.com.sg (K.M.H.); 8 Department of Pharmacology, Yong Loo Lin School of Medicine, National University of Singapore, Singapore 117600, Singapore; 9 Institution of Excellence, Vijnana Bhavan, University of Mysore, Manasagangotri, Mysore 570006, India

**Keywords:** formylchromone, STAT3 signaling, hepatocellular carcinoma, SHP-2, apoptosis

## Abstract

**Simple Summary:**

STAT3 acts as a potential tumor-promoting transcription factor that gets aberrantly activated in several types of human cancers and plays a crucial role in tumor progression and metastasis. STAT3 expression has been correlated with a dismal prognosis and poor survival. In this study, we have demonstrated that 3-formylchromone inhibits the STAT3 signaling in HCC cells by modulating SHP-2 expression. It also effectively diminished the tumor growth and subsequent reduction in metastasis in the HCC mouse model without exhibiting any major side effects.

**Abstract:**

Hepatocellular carcinoma (HCC) is one of the leading cancers that contribute to a large number of deaths throughout the globe. The signal transducer and activator of transcription 3 (STAT3) is a tumorigenic protein that is overactivated in several human malignancies including HCC. In the present report, the effect of 3-formylchromone (3FC) on the STAT3 signaling pathway in the HCC model was investigated. 3FC downregulated the constitutive phosphorylation of STAT3 and non-receptor tyrosine kinases such as JAK1 and JAK2. It also suppressed the transportation of STAT3 to the nucleus and reduced its DNA-binding ability. Pervanadate treatment overrode the 3FC-triggered STAT3 inhibition, and the profiling of cellular phosphatase expression revealed an increase in SHP-2 levels upon 3FC treatment. The siRNA-driven deletion of SHP-2 led to reinstate STAT3 activation. 3FC downmodulated the levels of various oncogenic proteins and decreased CXCL12-driven cell migration and invasion. Interestingly, 3FC did not exhibit any substantial toxicity, whereas it significantly regressed tumor growth in an orthotopic HCC mouse model and abrogated lung metastasis. Overall, 3FC can function as a potent agent that can display antitumor activity by targeting STAT3 signaling in HCC models.

## 1. Introduction

Hepatocellular carcinoma (HCC) originates in the liver and is one of the frontline causes of cancer-associated deaths globally [1,2,3]. According to the WHO, liver cancer stands fourth in terms of global cancer mortality with a death number of 830,000 in 2020 [4]. Liver cirrhosis due to hepatitis B/C infection, heavy alcohol consumption, hemochromatosis, obesity, and diabetes are the major risk factors of HCC [5,6]. Surgery, chemotherapy, immunotherapy, and targeted therapy are the available treatment approaches [7]. The delayed diagnosis of HCC results in poor long-term survival rates.

The signal transducer and activator of transcription (STAT) is a tumorigenic transcription factor that is present in the inactive monomeric form and can be activated by a wide array of ligands including cytokines, growth factors, and hormones [8,9,10,11]. The binding of ligands to their respective receptors on the plasma membrane results in the triggering of the activity of receptor tyrosine kinase (EGFR and PDGFR) or receptor-associated cytoplasmic nonreceptor tyrosine kinases (JAKs and Src) [12,13,14]. These upstream kinases phosphorylate STAT monomers at specific tyrosine residues, which leads to their dimerization and transportation to the nucleus to induce the transcription of target genes [15,16,17]. Among seven members of the STAT family, majorly the IL-6 family cytokines induce the phosphorylation of STAT3^Y705^, followed by subsequent dimerization and translocation, thus regulating its transcriptional activity [18,19,20]. Deregulated STAT3 activation has been reported in various human cancers including HCC and linked with the advancement of cancer, chemoresistance, poor clinical outcome, and decreased overall survival rate [21,22,23,24]. Therefore, the termination of the deregulated STAT3 cascade is an attractive therapeutic approach to counterbalancing the oncogenic and prosurvival effects of STAT3 in human cancers.

Chromones are the heterocyclic compounds bearing oxygen as a heteroatom in their structures and are widely present in the plant kingdom [25,26]. Chromone and its derivatives have exhibited a broad range of pharmacological properties including anticancer, antiviral, antimicrobial, and anti-inflammatory activities [27,28]. Among chromone derivatives, 3-formylchromone (3FC) and its derivatives have been reported to induce cytotoxicity in various cancer models by targeting diverse cellular targets such as STAT3, thymidine phosphorylase, DNA topoisomerase IIα, and the reversing of multidrug resistance [29,30]. The Schiff bases of 3FC were reported to inhibit thymidine phosphorylase, whereas 3FC was inactive [31]. Ko and colleagues reported that 3FC can display antiproliferative activity by impeding the STAT3 pathway in multiple myeloma cells [32]. In this report, we have investigated the impact of 3FC on STAT3 activation and subsequent signaling events in HCC cells and a preclinical orthotopic cancer model.

## 2. Materials and Methods

### 2.1. Cell Lines and Reagents

The information related to the procurement of cell lines, chemicals, and all the reagents has been given in our previous publications [33]. Antibodies against phospho-STAT3, phospho-JAK1, phospho-JAK2, caspase-8, caspase-9, MMP-9, STAT3, SHP-1, Src, JAK1, and JAK2 were obtained from Cell Signaling Technology (Danvers, MA, USA). Bcl-2, Bcl-xL, cyclin D1, survivin, Mcl-1, Bid, SHP-2, procaspase-3, and PARP were obtained from Santa Cruz Biotechnology (Santa Cruz, CA, USA). Primary and secondary antibodies were used in the dilutions of 1:1000 and 1:10,000, respectively. siRNAs of SHP-2 (sc-36488) and the control were obtained from Santa Cruz Biotechnology (Santa Cruz, CA, USA).

### 2.2. Immunocytochemistry

HCCLM3 cells were treated with 50 µM 3FC for 6 h, and the distribution of STAT3 was examined as elaborated earlier [34].

### 2.3. Western Blotting

HCC cells were incubated with 3FC as per the experimental conditions provided in the figures, protein expression levels were analyzed as reported earlier [35,36,37]. The densitometric analysis of Western blotting was performed using Image J software (Version 1.53n).

### 2.4. DNA Interaction Studies

A TransAM^TM^ ELISA kit (Active Motif, Carlsbad, CA, USA) was used to study the interaction between STAT3 with DNA as reported before [38,39].

### 2.5. STAT3-Luciferase Reporter Assay

A STAT3-dependent luciferase reporter gene expression assay was performed as reported earlier [40,41]. Briefly, 1 × 10^4^ Huh-7 cells were seeded in each well of 96-well plates and transfected with wild-type STAT3-responsive elements that were engineered with a luciferase gene or dominant-negative STAT3^Y705F^. After 24 h of transfection, the cells were incubated with 3FC for 6 h and then treated with epidermal growth factor (EGF) for 24 h. Thereafter, the cells were subjected to the lysis and the measurement of luciferase activity.

### 2.6. Transfection Experiments

The knockdown of SHP-2 was performed by siRNA transfection. The HCCLM3 cells (5 × 10^5^ cells/well) were transfected with SHP-2 siRNA using the NeonTM Transfection System (Invitrogen, Carlsbad, CA, USA). The HCCLM3 cells were transfected with SHP-2 siRNA (100 nM) or scrambled siRNA (100 nM) for 24 h, and the protein expression was analyzed using Western blotting.

### 2.7. Migration Assay

The effect of 3FC on cell migration was analyzed using an IBIDI culture insert (IBIDI GmbH), as reported previously [42].

### 2.8. Invasion Assay

Invasion assay was performed using a BD Biocoat^TM^ Matrigel^TM^ invasion chamber, as reported earlier [43].

### 2.9. Acute Toxicity Studies

Acute toxicity studies were performed on par with the protocols approved by the SingHealth Institutional Animal Use and Care Committee. For this purpose, eight-week-old male SCID mice were intraperitoneally injected with indicated doses of 3FC. The animals were monitored routinely for any unusual behavior, toxic symptom, change in the body weight, water, and feed intake up to day 8. The animals were sacrificed on the 8th day by cardiac puncture, and blood was collected to analyze kidney and liver functions.

### 2.10. In Vivo Orthotopic HCC Model

All the experimental protocols were on par with the protocols approved by the SingHealth Institutional Animal Use and Care Committee (protocol number: 2013/SHS/870). Eight-week-old male SCID mice were orthotopically impregnated with HCCLM3-Luc cell-originated tumors. When the photon count reached 10^6^, the animals were intraperitoneally administered with 3FC (100 mg/kg body weight; three doses per week) for four weeks. The tumor progression was continuously quantified based on bioluminescence signals. The mice were sacrificed by carbon-dioxide inhalation. Liver and lung tissues were collected and examined for metastasis using a Xenogen, IVIS 100 Bioluminescent Imaging System.

### 2.11. Statistical Analysis

Student’s t-test and one-way ANOVA were employed to measure statistical significance. For toxicity studies, a one-way ANOVA with Bonferroni multiple comparison test was carried out for the comparison among more than two experimental groups. For efficacy studies, an unpaired t-test with Welch’s correction was used to determine the significance between the two experimental groups. The data are presented as the mean ± SD. and error bars correspond to SD. *p* of <0.05 was considered statistically significant.

## 3. Results

### 3.1. 3FC Selectively Reduces Constitutive STAT3 Phosphorylation in HCC Cells

**Figure 1:**
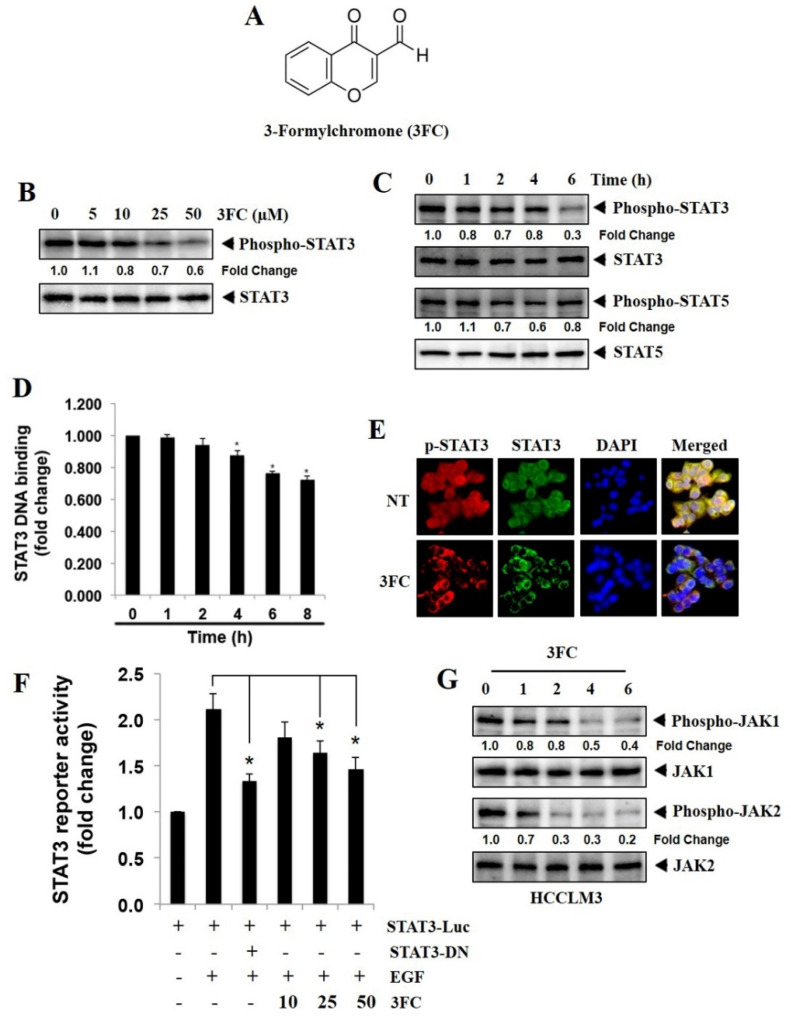
3-formylchromone (3FC) inhibits the activation of the signal transducer and activator of transcription 3 (STAT3). (**A**) Chemical structure of 3FC. (**B**) Effects of 3FC on STAT3 phosphorylation in HCCLM3 cells at different doses. HCCLM3 cells were incubated with various doses of 3FC for 6 h, followed by the preparation of cell lysate, resolution on an SDS-PAGE gel (7.5%), transfer onto the membrane, and probing for the detection of indicated proteins. (**C**) Effects of 3FC on phospho-STAT3, STAT3, phospho-STAT5, and STAT5 in HCCLM3 cells at different time points using Western blotting. 3FC (50 µM)-treated HCCLM3 cells were used for Western blotting to visualize the levels of phospho-STAT3, STAT3, phospho-STAT5, and STAT5. (**D**) STAT3–DNA binding as a function of time. Nuclear extracts prepared from 3FC (50 µM)-treated HCCLM3 cells were used for STAT3–DNA binding studies. The experiment was performed in triplicates, and the SDs between the triplicates are indicated. * *p* < 0.05. (**E**) Distributions of STAT3 in the nuclei and cytoplasms of the control and the 3FC (50 µM for 6 h)-treated HCCLM3 cells using immunocytochemistry. (**F**) STAT3-driven reporter gene expression as a function of the dose. A STAT3-luciferase (STAT3-Luc) plasmid was introduced into Huh-7 and grown for 24 h with subsequent treatment with 3FC at indicated doses and induction with epidermal growth factor (EGF; 100 ng/mL). Thereafter, cells were lysed, and the luciferase activity was measured in the whole-cell extracts. (**G**) Impacts of 3FC on the constitutive activation of JAK1 and JAK2 at different time points using Western blotting. 3FC (50 µM)-treated HCCLM3 cells used for Western blotting to analyze the levels of indicated proteins. The experiment was performed in triplicates, and the SDs between the triplicates are indicated. * *p* < 0.05. The uncropped western blot figures can be accessed as supplementary file.

As the HCCLM3 cell line expresses substantial levels of constitutively active STAT3, the potential of 3FC to inhibit the deregulated STAT3 phosphorylation in these tumor cells was first examined. The structure of 3FC is given in Figure 1A. For this purpose, HCCLM3 cells were incubated with indicated doses of 3FC at different time points, and the effects of 3FC on STAT3 phosphorylation at various time durations were examined. 3FC significantly mitigated STAT3 phosphorylation in a dosage- and duration-dependent fashion without affecting the levels of total STAT3 (Figure 1B,C). The effects of 3FC on the phosphorylation of STAT5 in HCCLM3 cells at different time points were also evaluated. 3FC had a minimal effect on STAT5 phosphorylation (Figure 1C), indicating its specificity towards the inhibition of STAT3 phosphorylation.

### 3.2. 3FC Decreases the DNA-Binding Potential of STAT3 in HCC Cells

The nuclear extracts obtained from the 3FC-treated cells and the control cells were incubated with an oligonucleotide containing a STAT3-specific DNA probe to quantify the ability of STAT3 to interact with DNA. 3FC treatment significantly downregulated the DNA interaction ability of STAT3 in a duration-dependent fashion (Figure 1D).

### 3.3. 3FC Downmodulates the Nuclear Translocation of STAT3 in HCCLM3 Cells

The localization of STAT3 in the cytoplasm and nuclei of the control and the 3FC-treated HCCLM3 cells were analyzed using immunostaining methods. The distributions of STAT3 were observed in the cytoplasm and nucleus of the control cells, and the treatment with 3FC significantly reduced the nuclear STAT3 (Figure 1E), suggesting the interference of 3FC in the STAT3 pathway in HCC cells.

### 3.4. 3FC Counteracts STAT3-Driven Luciferase Gene Expression in HCC Cells

A STAT3-Luc plasmid was used for transfection experiments. The transfected Huh-7 cells were treated with indicated doses of 3FC and induced with EGF, as they expressed relatively lower levels of constitutively active STAT3 to measure the luciferase activity. We found that EGF stimulation significantly increased the STAT3-directed luciferase gene expression. The treatment with dominant-negative STAT3 reversed this effect, suggesting its specificity towards the target. 3FC treatment reduced the STAT3-driven reporter gene expression in a concentration-dependent fashion (Figure 1F).

### 3.5. 3FC Decreases the Constitutive Activation of JAK Proteins in HCC Cells

The impacts of 3FC on the constitutive activation of JAK1 and JAK2 were examined. In HCCLM3 cell lines, it was found that 3FC substantially suppressed the persistent phosphorylation of both proteins in a time-dependent fashion (Figure 1G).

### 3.6. Pervanadate Reverses 3FC-Driven STAT3 Inhibition in HCC Cells

**Figure 2:**
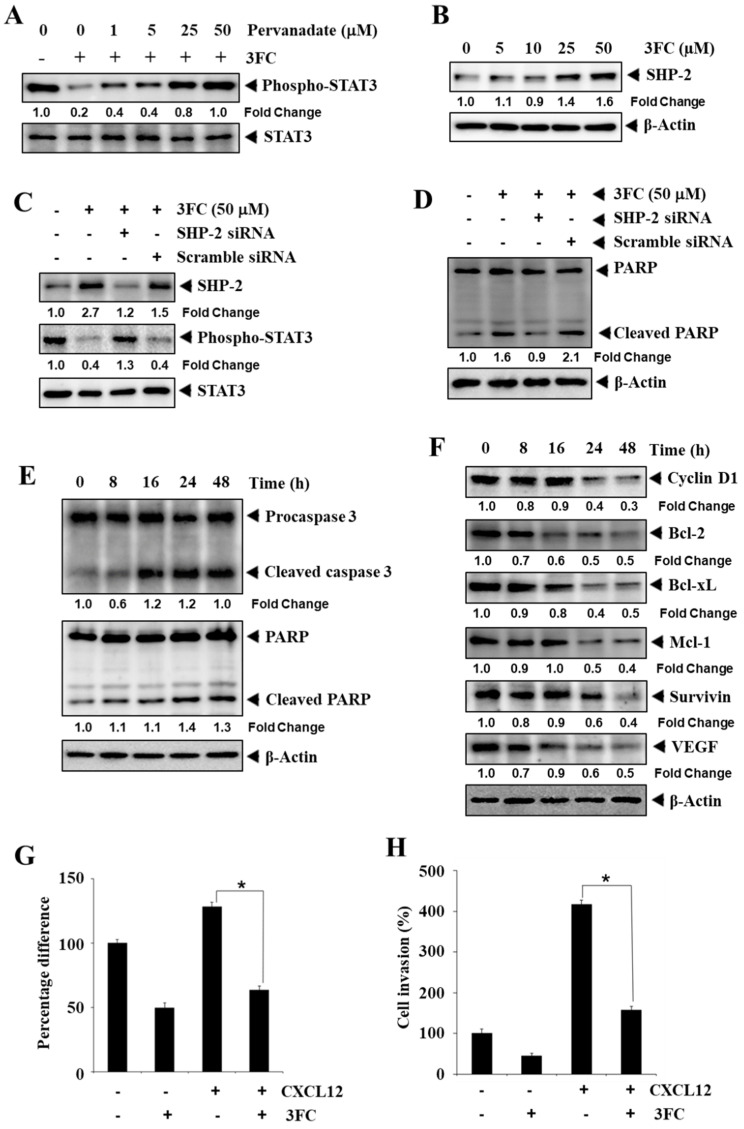
SHP2 is involved in 3FC-mediated STAT3 inhibition. (**A**) Effects of pervanadate with different concentrations on the STAT3-inhibitory activity of 3FC using Western blotting. Pervanadate and/or 3FC treated HCCLM3 cells were used for Western blotting to visualize the levels of phospho-STAT3 and STAT3. (**B**) Impacts of 3FC with different concentrations on SHP-2 expression in HCCLM3 cells using Western blotting. 3FC-treated HCCLM3 cells were used for Western blotting to analyze the levels of SHP-2. (**C**) Effects of 3FC (50 µM for 6 h) on STAT3 activation using scrambled siRNA- or SHP-2 siRNA-transfected HCCLM3 cells and Western blotting. (**D**) Effects of 3FC (50 µM for 24 h) on PARP cleavage using scrambled siRNA- or SHP-2 siRNA-transfected HCCLM3 cells and Western blotting. (**E**) Effects of 3FC on the levels of the indicated apoptosis-modulating proteins (procaspase-3, PARP, and cleaved fragment of PARP) at different time points using Western blotting. (**F**) Effects of 3FC on the levels of cyclin D1, Bcl-2, Bcl-xL, Mcl-1, survivin, and VEGF proteins at different time points using Western blotting. (**G**) Impact of 3FC on the mobility of HCCLM3 cells. A cell culture dish setup was established as described in the methods. HCCLM3 cells were propagated in the culture dish, and a cell-free gap was created. These cells were induced with a chemokine (CXCL12, 100 ng/mL) and treated with 50 μM 3FC. Thereafter, cell mobility was measured, and the width of the cell-free gap and the graph were plotted. The experiment was performed in triplicates, and the SDs between the triplicates are indicated. * *p* < 0.05. (**H**) Impact of 3FC on the invasion of HCCLM3 cells. An invasion assay was performed as indicated earlier. HCCLM3 cells were induced with CXCL12, the anti-invasive ability of 3FC was measured, and the graph was plotted. The experiment was performed in triplicates, and the SDs between the triplicates are indicated. * *p* < 0.05. The uncropped western blot figures can be accessed as supplementary file.

HCCLM3 cells were treated with pervanadate, a phosphatase inhibitor, and 3FC to examine their effects on the phosphorylation of STAT3. Interestingly, the treatment of pervanadate reversed the STAT3-inhibitory activity of 3FC in a concentration-dependent fashion (Figure 2A).

### 3.7. 3FC Induces the Expression of SHP-2 to Impart STAT3 Inhibition in HCC Cells

Next, the impact of 3FC on SHP-2 expression in HCCLM3 cells was examined using Western blotting analysis. The incubation of HCCLM3 cells with 3FC induced the expression of SHP-2 (Figure 2B). Subsequently, SHP-2 was deleted from HCCLM3 cells using SHP-2 siRNA followed by 3FC treatment, and these cells were examined for the expression of phosphorylated STAT3. 3FC treatment alone significantly induced SHP-2 expression with a decline in STAT3 phosphorylation (Figure 2C). SHP-2-transfected and 3FC-treated cells showed a reduced expression level of SHP-2 and elevated STAT3 phosphorylation (Figure 2C). Scrambled RNA-transfected and 3FC-treated cells displayed an increased SHP-2 expression level with a clear decrease in STAT3 phosphorylation. In addition, the knockdown of SHP-2 resulted in a reduction of 3FC-driven PARP cleavage, whereas 3FC significantly induced PARP cleavage in control cells (Figure 2D).

### 3.8. 3FC Induces the Cleavage of PARP and Procaspase-3 in HCC Cells

HCCLM3 cells were treated with 3FC for the indicated duration, and the expression levels of procaspase-3 and PARP were analyzed. 3FC downmodulated the levels of procaspase-3 and PARP and increased the level of the cleaved fragment of PARP (Figure 2E), thus suggesting that 3FC can induce apoptosis in HCC cells.

### 3.9. 3FC Mitigates the Expression of Apoptosis-Associated Proteins

STAT3 transcriptionally modulates the expression of genes associated with apoptosis [44]. Therefore, the effects of 3FC on the levels of cyclin D1, Bcl-2, Bcl-xL, Mcl-1, survivin, and VEGF proteins at different time points were evaluated using Western blotting. 3FC mitigated the expression levels of all the tested proteins in a duration-dependent fashion, confirming the STAT3 inhibitory activity of 3FC (Figure 2F). β-actin was used as a loading control.

### 3.10. 3FC Displays Antimigratory and Anti-Invasive Potential towards HCC Cells

The impact of 3FC on the mobility of HCCLM3 cells was analyzed using a special culture dish. The cells were cultured in a culture disc, which created a cell-free space of approximately 500 µm, and these cells were treated with 3FC alone and in combination with CXCL12. The treatment with CXCL12 significantly induced the migration of HCCLM3 cells, and 3FC significantly counteracted the CXCL12-induced cell migration (Figure 2G). Next, the impact of 3FC on the invasion of HCCLM3 cells was investigated using transwell chambers. The treatment with CXCL12 increased the invasion and co-treatment of 3FC, along with CXCL12, significantly downregulated the number of invaded cells (Figure 2H). These results suggested that 3FC possessed antimigratory and anti-invasive potential towards HCC cells.

### 3.11. 3FC Does Not Display Toxicity in In Vivo Experiments

**Figure 3:**
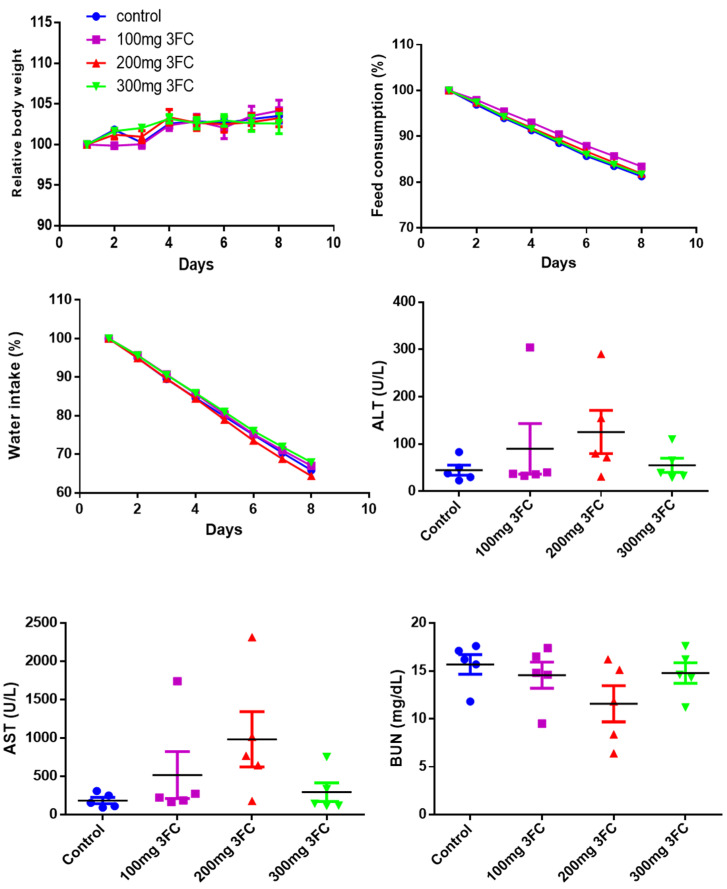
Levels of serum biochemical markers such as alanine aminotransferase (ALT), aspartate aminotransferase (AST), and blood urea nitrogen (BUN) in the control group and the groups treated with 3FC at the intraperitoneal administration of 100, 200, and 300 mg/kg body weight. Eight-week-old male mice (*n* = 5) were intraperitoneally administered with indicated doses of 3FC, and the changes in body weight, food consumption, and water intake were analyzed. After 8 days, animals were sacrificed, and blood was collected and analyzed for the activity of ALT, AST, and BUN to study the toxicity profile of 3FC. The *p*-value was non-significant among all groups.

Acute toxicity studies were performed to determine the possible adverse effects of 3FC in SCID mice. Single doses at indicated concentrations of 3FC were intraperitoneally administered to different groups of SCID mice, and toxic symptoms and mortality were examined. No mortality was observed throughout the study duration in the 3FC-treated animals. Besides, the 3FC-administered animals did not present significant changes in body weight, feed, and water consumption relative to the control animals (Figure 3). There were no major changes in the levels of serum biochemical markers such as alanine aminotransferase (ALT), aspartate aminotransferase (AST), and blood urea nitrogen (BUN), compared to in the vehicle-treated animals (Figure 3).

### 3.12. 3FC Impedes Tumor Growth and Metastasis In Vivo

**Figure 4:**
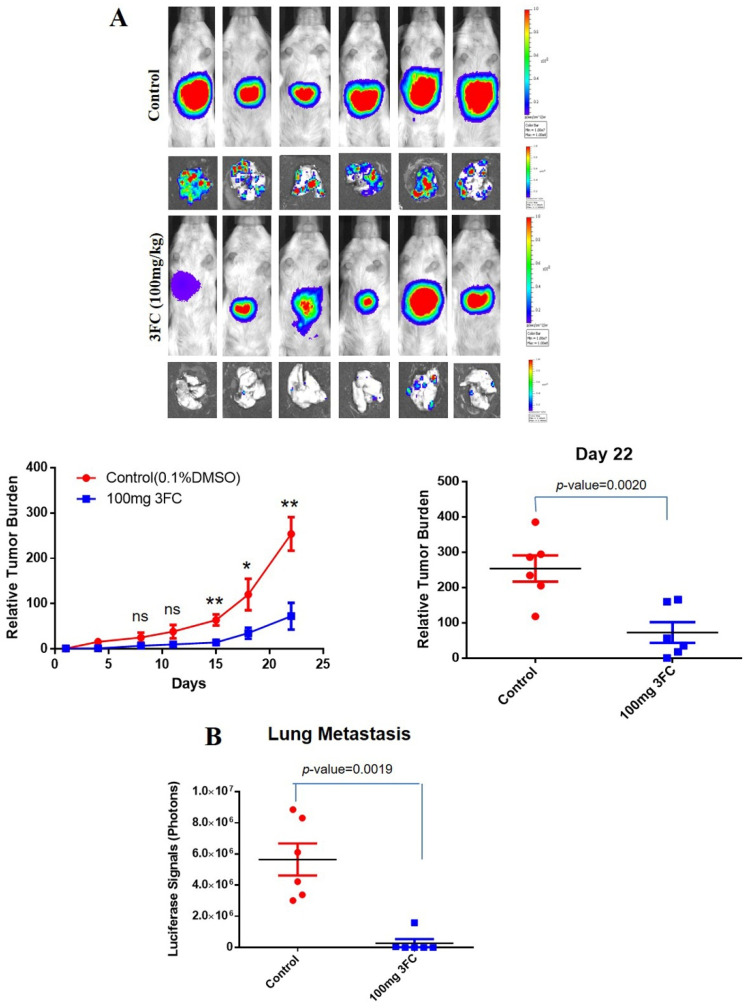
(**A**) Growth-inhibitory effect of 3FC on HCCLM3-Luc cells implanted in mice. (**B**) Lung metastasis was measured by quantifying the bioluminescence signals from the secondary tumor site. * *p* < 0.05, ** *p* < 0.01. The tumors generated using HCCLM3-Luc cells were orthotopically implanted into 8-week-old male SCID mice (*n* = 6), and tumor progression was analyzed. When the tumor reached 10^6^, animals were intraperitoneally administered with 3FC (100 mg/kg body weight, three doses per week) for four weeks, and the growth of the tumor was examined by measuring the photon count.

An orthotopic HCC mouse model was established as elaborated in methodology, and the growth-inhibitory potential of 3FC was investigated. The intraperitoneal (i.p.) administration of 3FC (100 mg/kg body weight) significantly reduced the tumor growth (Figure 4A) with a corresponding decline in metastasis to lungs compared with the control animals (Figure 4B). The tumor expansion/regression was recorded by quantifying the photon counts prior to 3FC treatment and during the termination of the study, as described in our previous reports [45,46]. A marginal surge in body weight was observed in the animals treated with 3FC compared to control group animals.

## 4. Discussion

STAT3 is one of the widely-studied transcription factors that encourage tumor initiation and progression, antiapoptosis, and metastasis [47,48,49]. STAT3 is persistently activated in human malignancies including liver cancer, breast cancer, prostate cancer, ovarian cancer, kidney cancer, and head and neck cancer [50,51,52,53]. The persistent activation of STAT3 is attributed to the disruption of negative modulators of STAT3 signaling, somatic mutations in STAT3, enhanced activity of upstream kinases, and establishment of the positive feedback loop in the tumor microenvironment [54,55,56,57]. Notably, studies have demonstrated the general increase in the levels of STAT3 in HCC tumor tissue samples and activated nuclear STAT3 was observed in approximately 60% of HCC samples, which was correlated with malignant progression and negative prognosis [58,59,60]. Besides, the elevated expression of phosphorylated STAT3 in HCC tissues has been reported to negatively contribute to prognosis after surgical procedures [59,61]. The overexpression of STAT3 is entangled with the advancement of the tumor stage and has been identified as an important biomarker for prognosis prediction in solid tumors [62]. Therefore, we have focussed on the investigation of STAT3-signaling inhibitory activity of 3FC in cell-based assays and preclinical HCC models.

The Western blotting results suggested that 3FC diminished the constitutive phosphorylation of STAT3^Y705^. Studies have demonstrated that STAT3 undergoes constitutive phosphorylation at Tyr-705 (STAT3^Y705^) by autocrine or paracrine signaling in the tumor microenvironment [63,64]. The activated STAT3 is dimerized via the SH2 domain and translocated into the nucleus. Therefore, we hypothesized that the inhibition of STAT3 phosphorylation by 3FC interfered with dimerization and restricted its translocation into the nucleus. To confirm this, the distributions of STAT3 in the nuclear and cytoplasmic compartments were analyzed using immunocytochemistry. The results showed the decline in nuclear STAT3 upon 3FC treatment, indicating that 3FC may restrict the STAT3 localization in the nucleus. However, unphosphorylated STAT3 can also interact with unphosphorylated NF-κB to modulate the expression of some nuclear genes [65]. Although 3FC reduced the nuclear STAT3 and its DNA interaction capability, the inhibition of phosphorylation is crucial in driving tumor cell proliferation and malignant progression [66]. In the pioneering studies, Src-transformed cells displayed a deregulated activity of STAT3, and the introduction of dominant-negative STAT3 suppressed transformation [67,68].

STAT3 modulates the transcription of genes associated with oncogenesis, antiapoptosis, cell cycle progression, metastasis, and angiogenesis [69,70,71]. In the 3FC-treated cells, the potential of nuclear STAT3 to associate with DNA was analyzed, and a decline in the DNA interaction potential was noted. These results demonstrated that 3FC interfered with the transcriptional activity of STAT3. To confirm this, the expression levels of antiapoptotic genes that are under the transcriptional control of STAT3 were investigated. 3FC markedly reduced the expression levels of cyclin D1, Bcl-2, Bcl-xL, Mcl-1, survivin, and VEGF proteins [72]. This suggested that the transcriptional function of STAT3 was abrogated in the tested cell lines. STAT3 is primarily activated by upstream proteins such as JAKs and Src [73,74,75]. 3FC counteracted the constitutive activation of JAK1 and JAK2, indicating that inhibition of STAT3 is due to the suppression of the activity of upstream kinases.

SHP-1, SHP-2, PTP1B, PTPε, and TC-PTP are some of the major phosphatases that modulate the phosphorylation status of JAK-STAT signaling pathway proteins [32,75,76]. Pervanadate is a phosphatase inhibitor, and the co-treatment of HCC cells with 3FC and pervanadate resulted in the reversing of 3FC-directed STAT3 inhibition. This observation suggested that 3FC modulates the expression or activity of a particular phosphatase to regulate STAT3 signaling in HCC cells. We have previously demonstrated that some natural and synthetic compounds impede STAT3 signaling through the upregulation of the expression levels of different phosphatases [76,77]. We found a substantial upregulation in expression of SHP-2 upon 3FC treatment. Structurally, SHP-2 has a phosphatase domain and two SH2 domains. Functionally, SHP-2 interacts with the phosphotyrosine of the target protein (such as JAKs, Src family kinases, and growth factor receptors) through the SH2 domain and dephosphorylates its substrate via phosphatase activity. SHP-2 is one of the major phosphatases involved in the downregulation of the STAT3 signaling pathway. It may be interpreted that 3FC induces the expression of SHP-2 to reduce the phosphorylation of STAT3. Additionally, Chong and colleagues demonstrated that IL-6 triggers STAT3 activation and drives the transcription of PRL-3 (an oncogenic phosphatase overexpressed in multiple myeloma) [78]. PRL-3 protein promotes STAT3 phosphorylation by deactivating SHP-2, thereby framing a feedforward loop in multiple myeloma [78]. Therefore, it may be speculated that PRL-3 or some other synonymous phosphatase is involved in establishing the link between the counterbalancing roles of STAT3 and SHP-2 in the HCC model. The protein inhibitor of activated STAT (PIAS) is a negative modulator of STAT family proteins. In addition to the induction of PTPs, 3FC has been described to repress STAT3 activity through the expression of PIAS3 in multiple myeloma cells [32]. The STAT3-dependent luciferase expression studies also supported the findings of previous experiments.

STAT3 also controls the expression of MMPs, Snail, and Twist, which can also regulate the metastatic phenotype [79,80], and 3FC treatment reduced the cell migration and invasion of HCC cells. Multiple studies have shown that the overexpression of CXCR4 is observed in cancers and is associated with cell migration and invasion. CXCL12 acts as a ligand for CXCR4 and plays a crucial role in autocrine/paracrine signaling in several cancers. Hence, we determined the effect of 3FC on the invasion and migration of HCC cells, which were under the influence of CXCL12. 3FC significantly reduced the CXCL12-driven cell motility in the tested assay systems. Some of the proteins encoded by STAT3 target genes (such as MMP-2, MMP-9, and Twist) were involved in the promotion of cell migration and invasion. Therefore, the observed antimigratory and anti-invasive potential of 3FC could be due to the inhibition of STAT3 activation.

Finally, 3FC displayed a good safety profile without toxicity up to 300 mg/kg body weight. No alteration in physical parameters and hematocrit was observed, suggesting that 3FC has no notable toxicity when administered intraperitoneally. The oral administration of 3FC did not display significant toxicity at the concentration of 25 mg/kg body weight in Wistar rats [81]. 3FC showed good antitumor potential against the orthotopic HCC tumor mice model and significantly decreased lung metastasis. Ilamathi and colleagues observed similar salubrious effects of 3FC towards nitrosodiethylamine-induced hepatocellular carcinogenesis in a rodent model [81].

Although 3FC has been shown to suppress STAT3 signaling and induce growth inhibition in cancer cell lines in an in vivo model, earlier studies have demonstrated that 3FC can also modulate functions of many cancer-promoting proteins (such as thymidine phosphorylase and DNA topoisomerase IIα). Moreover, 3FC also elevated the expression of SHP-2. Earlier studies also propose that PRL-3 (a phosphatase) interacts with SHP-2 to diminish its activity. Additionally, STAT3 signaling can display crosstalk with NF-κB, p53, Smad3, and many more signaling pathways, and the abrogation of STAT3 activity may alter all these associated pathways. Therefore, we speculate that 3FC does not induce anticancer activity solely through the inhibition of the STAT3 cascade and the observed antioncogenic effects are at least partly mediated via the mitigation of the STAT3 pathway.

## 5. Conclusions

Overall, 3FC has been demonstrated as a novel pharmacological agent that induces apoptosis by impeding STAT3 activation in HCC cell lines and preclinical models. 3FC induced the expression of SHP-2 to counteract the activation of STA3 signaling. It also impeded the lung metastasis of HCC cells in an in vivo mouse model. Thus, 3FC is presented as a novel abrogator of the STAT3 pathway in HCC cell lines and preclinical models.

## Data Availability

All data are freely available with this article.

## Supplementary Materials

The following supporting information can be downloaded at: https://www.mdpi.com/article/10.3390/biology11010029/s1, File S1: The uncropped western blot figures.
