# Supplementary material for: 3-Formylchromone Counteracts STAT3 Signaling Pathway by Elevating SHP-2 Expression in Hepatocellular Carcinoma
Source: Biology (Basel). 2021 Dec 26;11(1):29. doi: 10.3390/biology11010029 (PMC8773260; doi:10.3390/biology11010029)
Supplement: Supplementary file 1 [file biology-11-00029-s001.zip › biology-1519533-supplementary.pptx]

## Slide 1
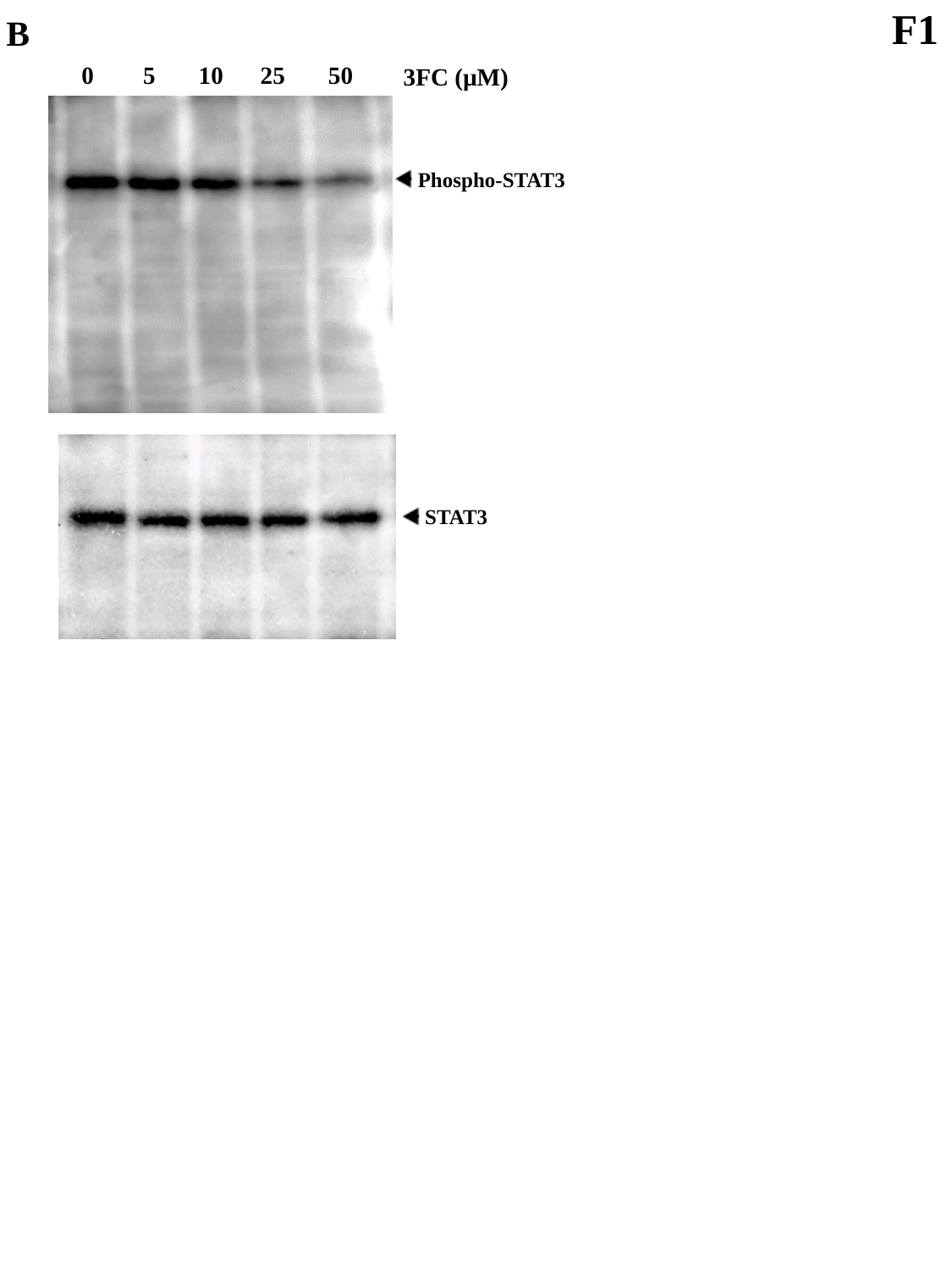

F1
B
 0 5 10 25 50
3FC (µM)
Phospho-STAT3
STAT3

## Slide 2
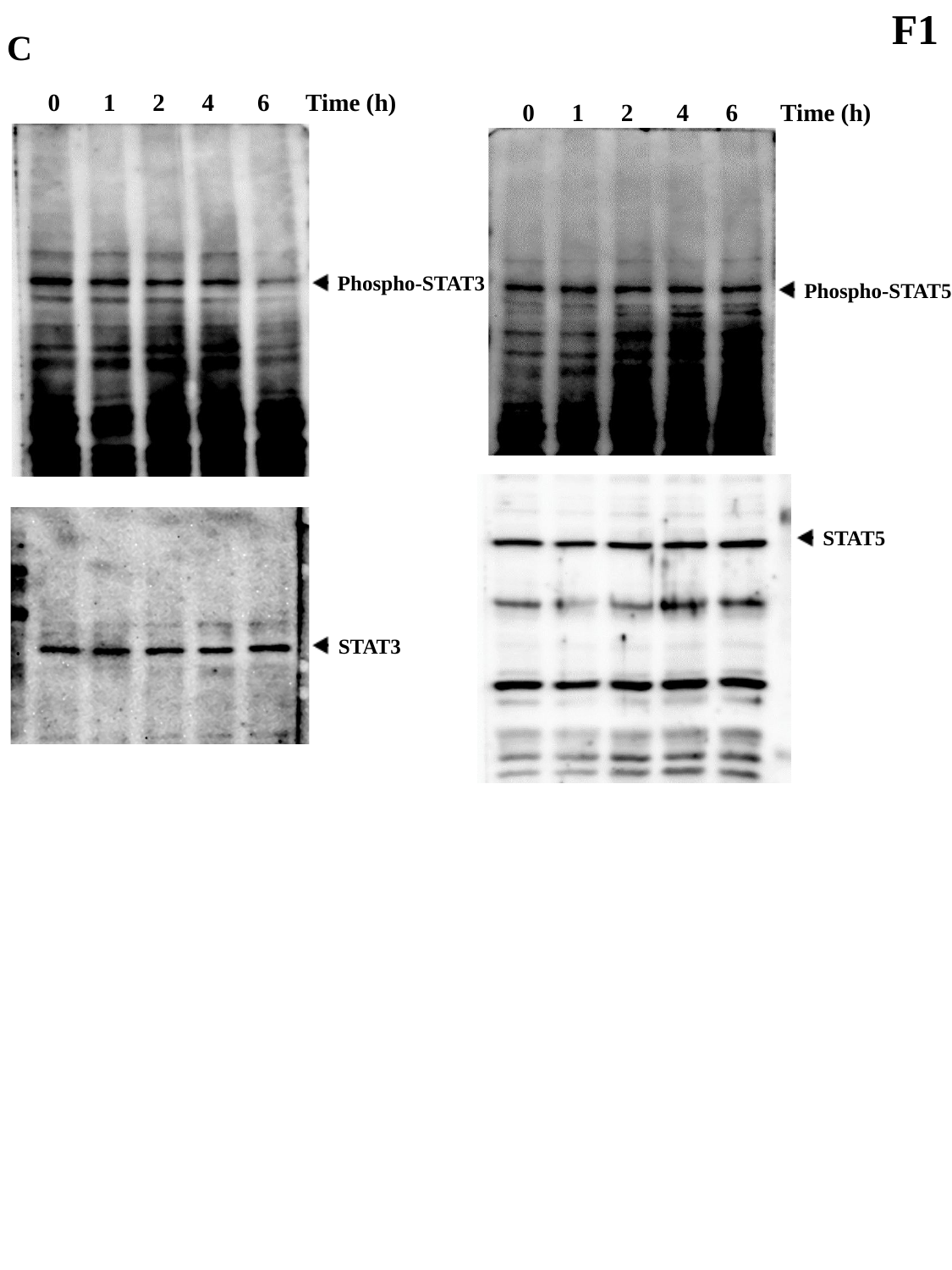

F1
C
Time (h)
 0 1 2 4 6
Time (h)
 0 1 2 4 6
Phospho-STAT3
Phospho-STAT5
STAT5
STAT3

## Slide 3
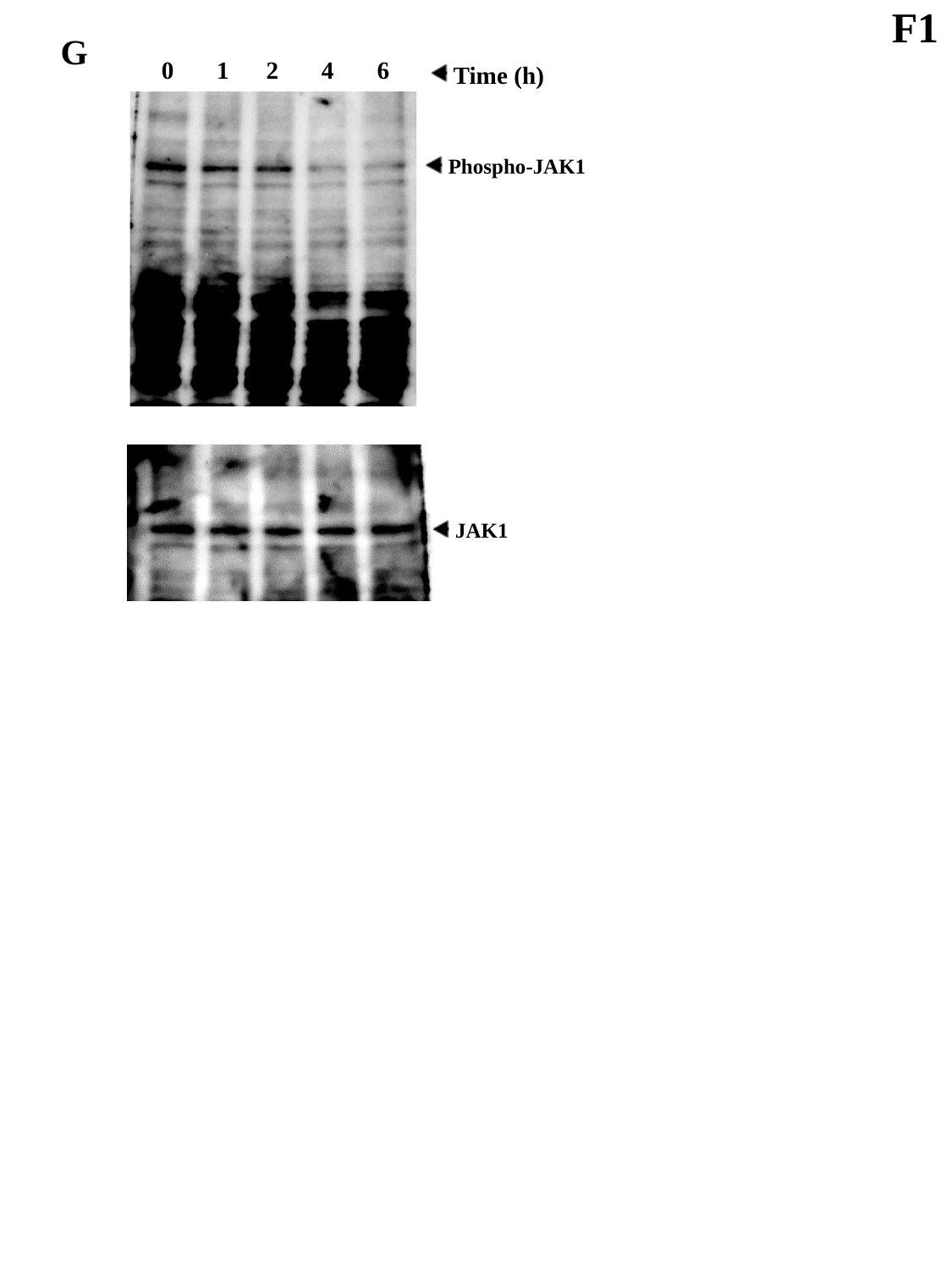

F1
G
 0 1 2 4 6
Time (h)
Phospho-JAK1
JAK1

## Slide 4
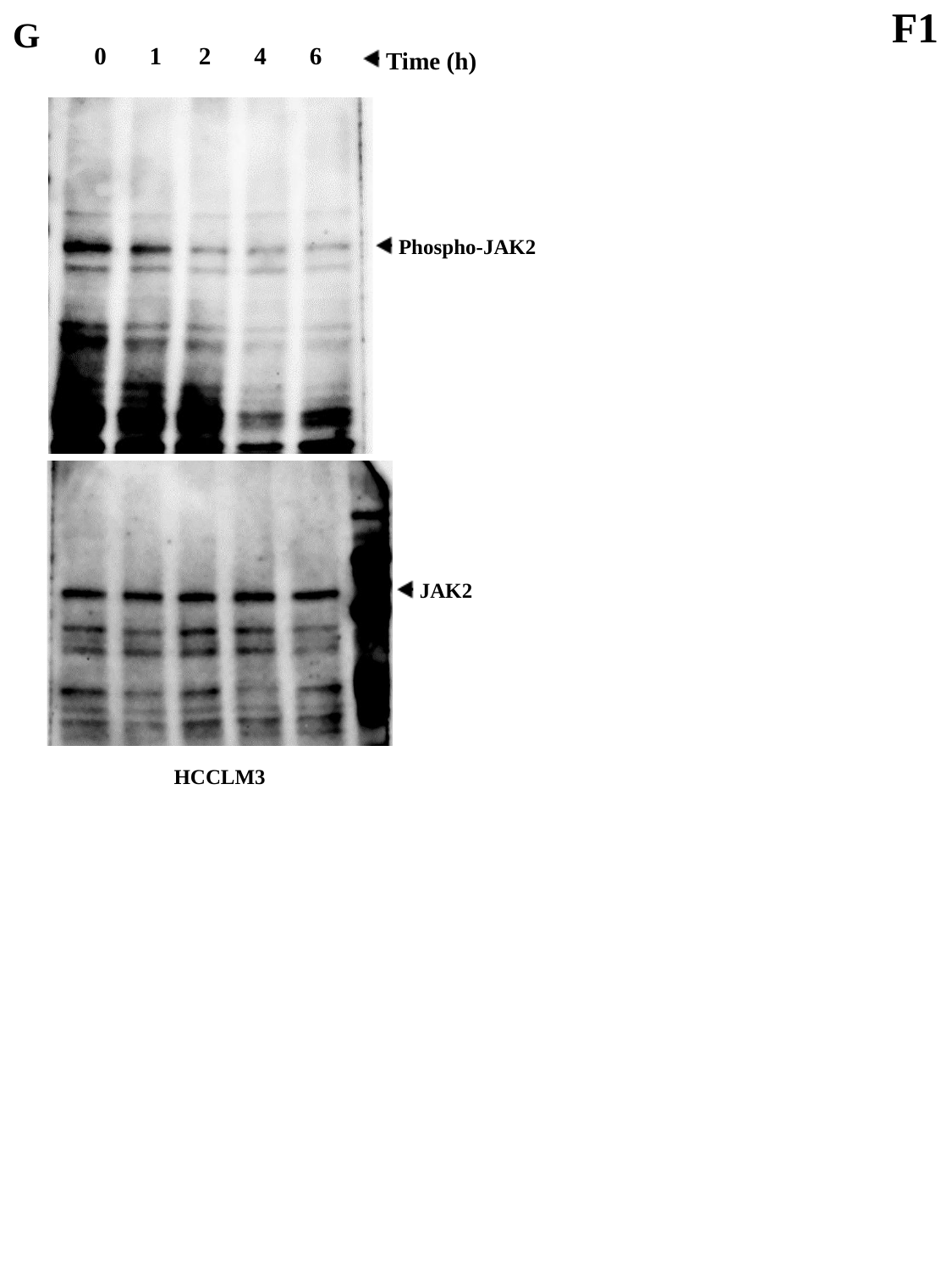

F1
G
 0 1 2 4 6
Time (h)
Phospho-JAK2
JAK2
HCCLM3

## Slide 5
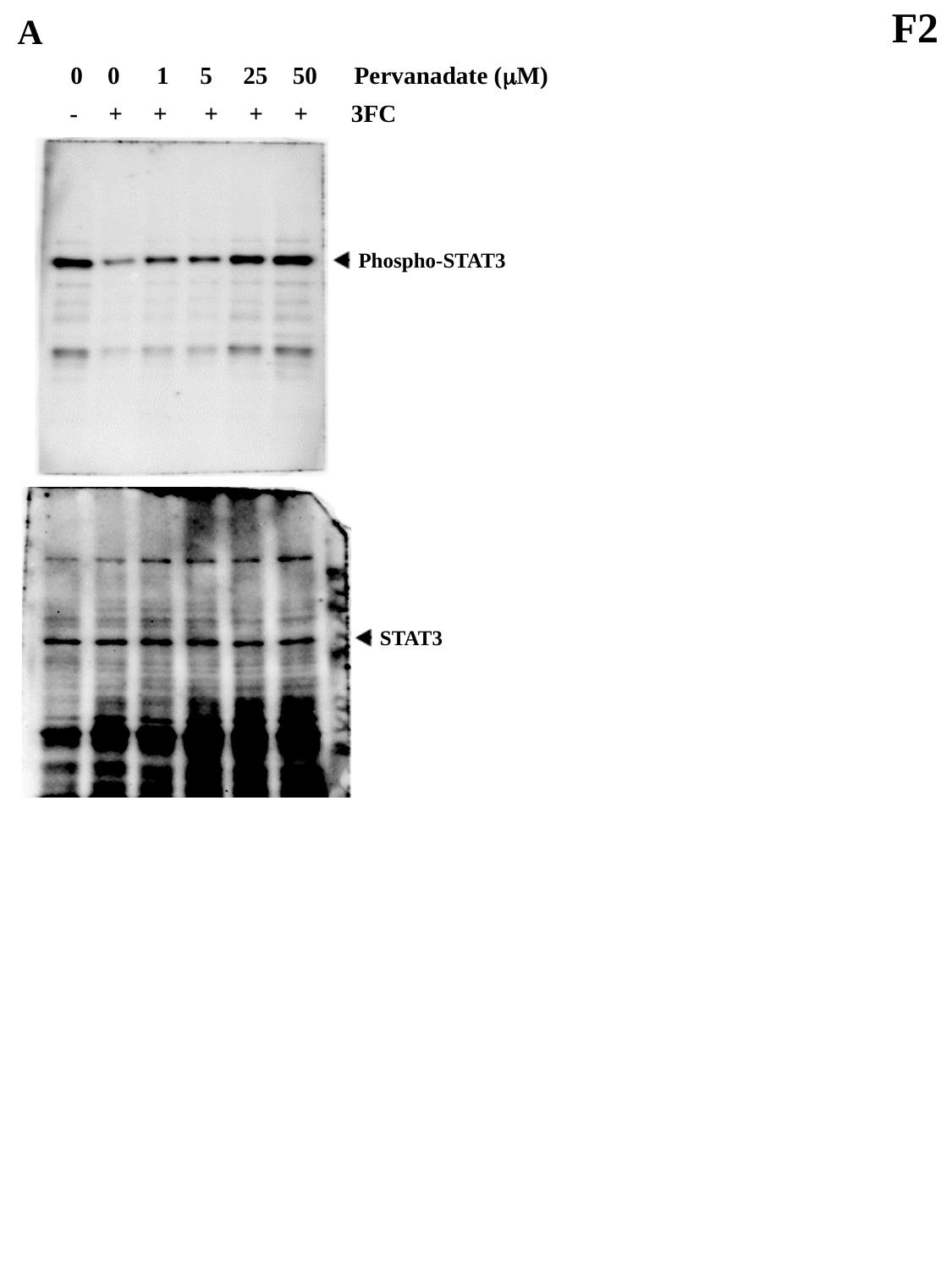

F2
A
 0 0 1 5 25 50 Pervanadate (μM)
 - + + + + + 3FC
Phospho-STAT3
STAT3

## Slide 6
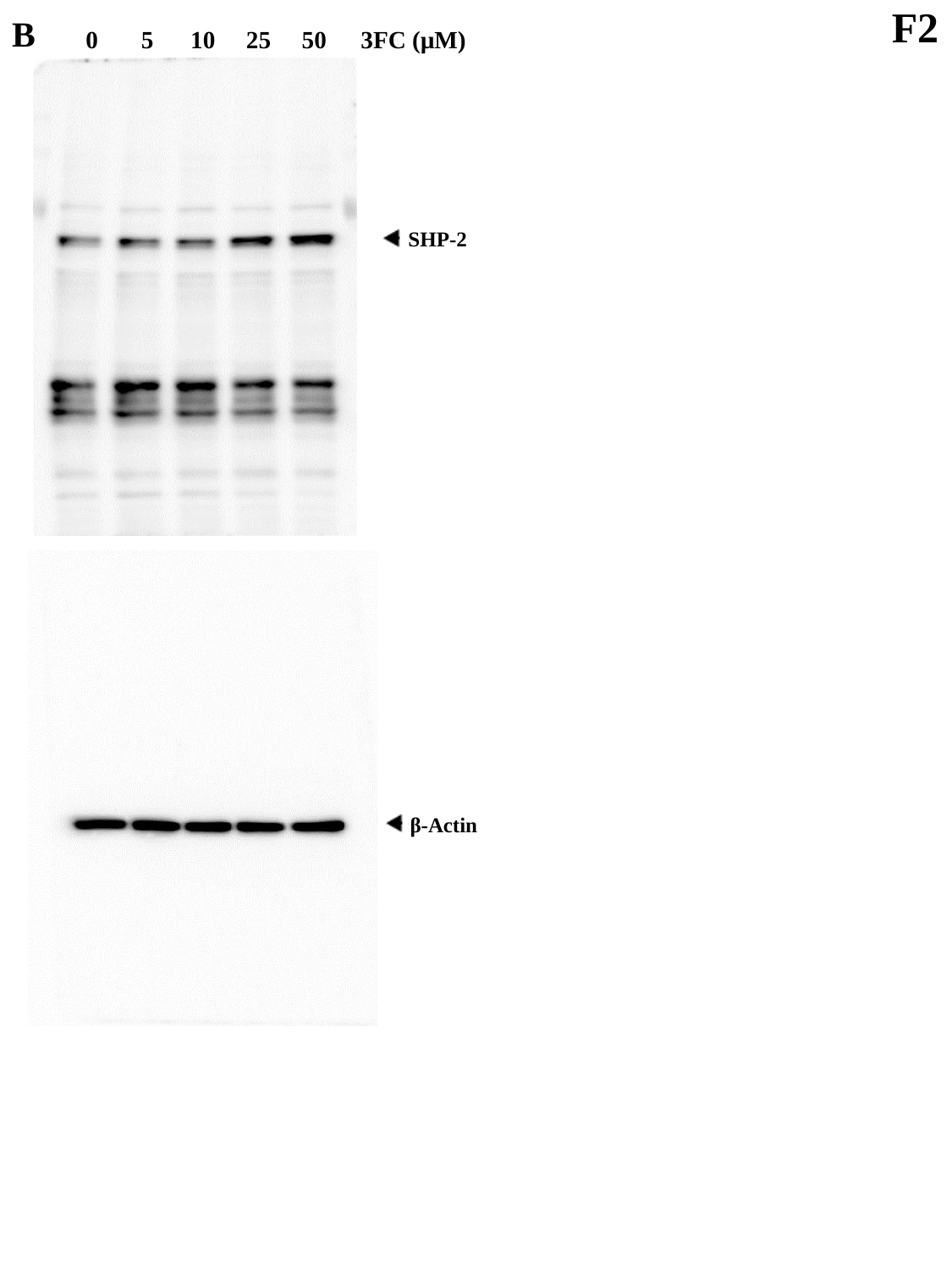

F2
B
 0 5 10 25 50
3FC (µM)
SHP-2
β-Actin

## Slide 7
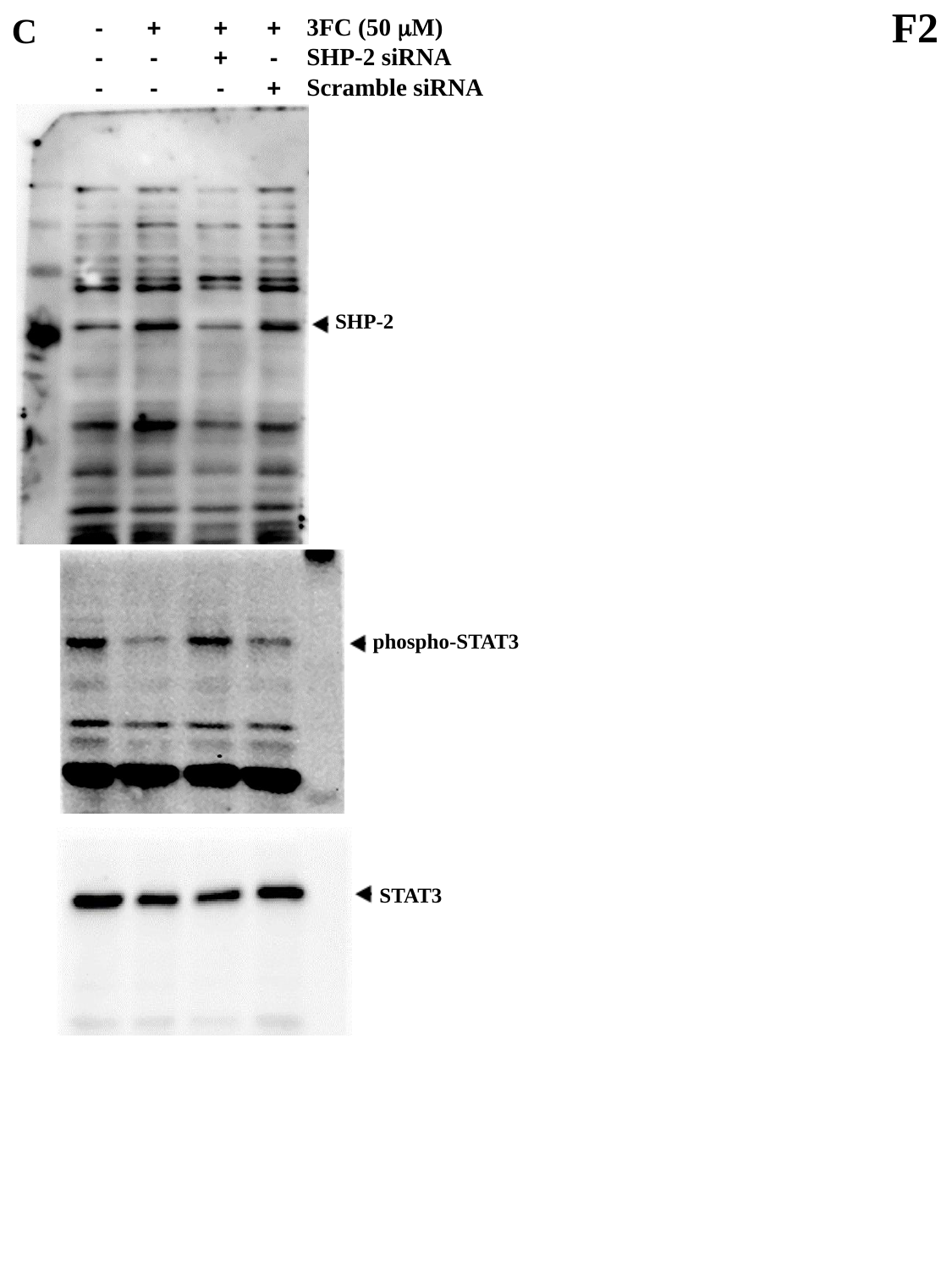

F2
C
3FC (50 mM)
-
+
+
+
SHP-2 siRNA
-
-
+
-
Scramble siRNA
-
-
-
+
SHP-2
phospho-STAT3
STAT3

## Slide 8
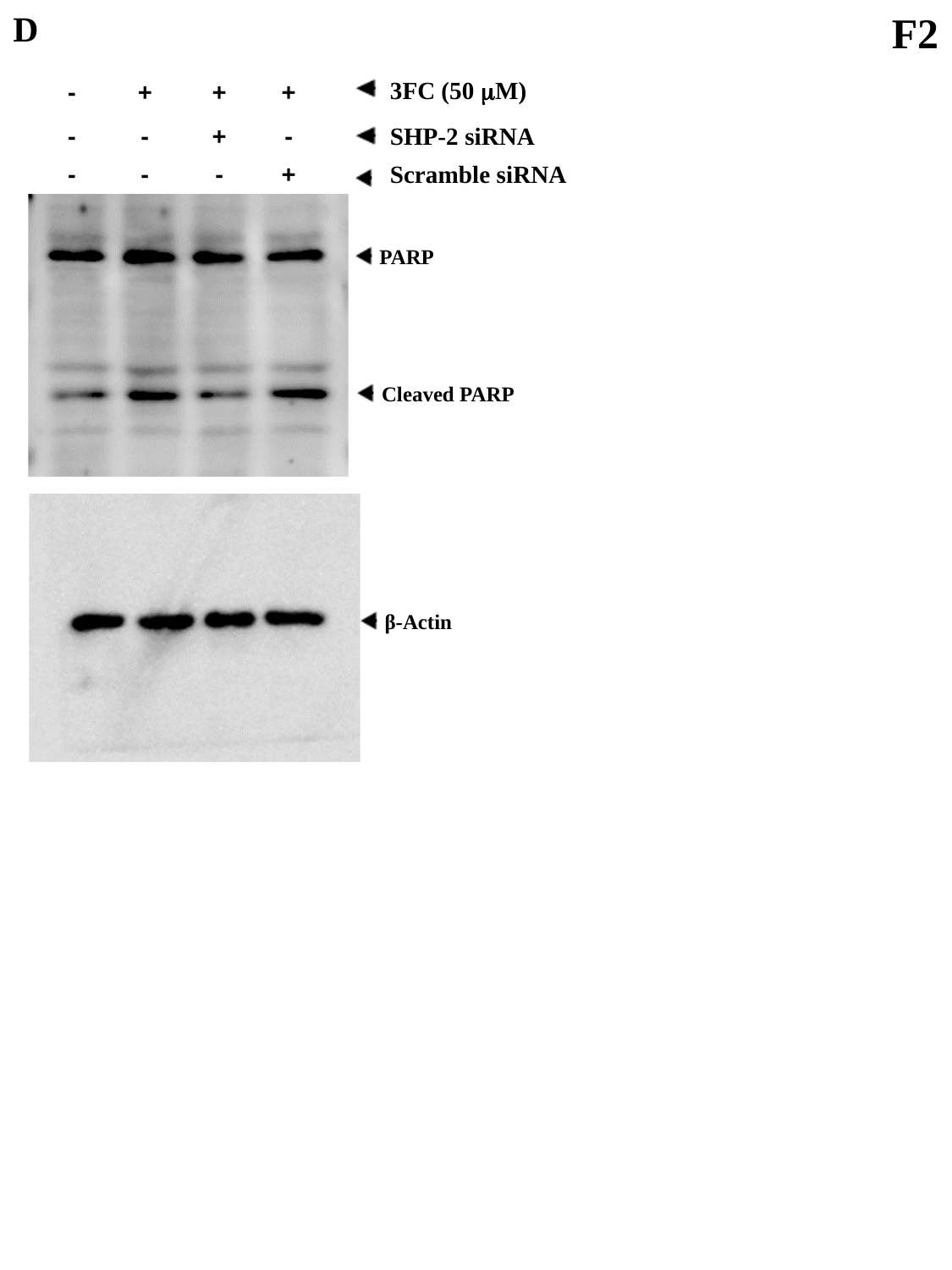

D
 F2
3FC (50 mM)
-
+
+
+
-
-
+
-
SHP-2 siRNA
-
-
-
+
Scramble siRNA
PARP
Cleaved PARP
β-Actin

## Slide 9
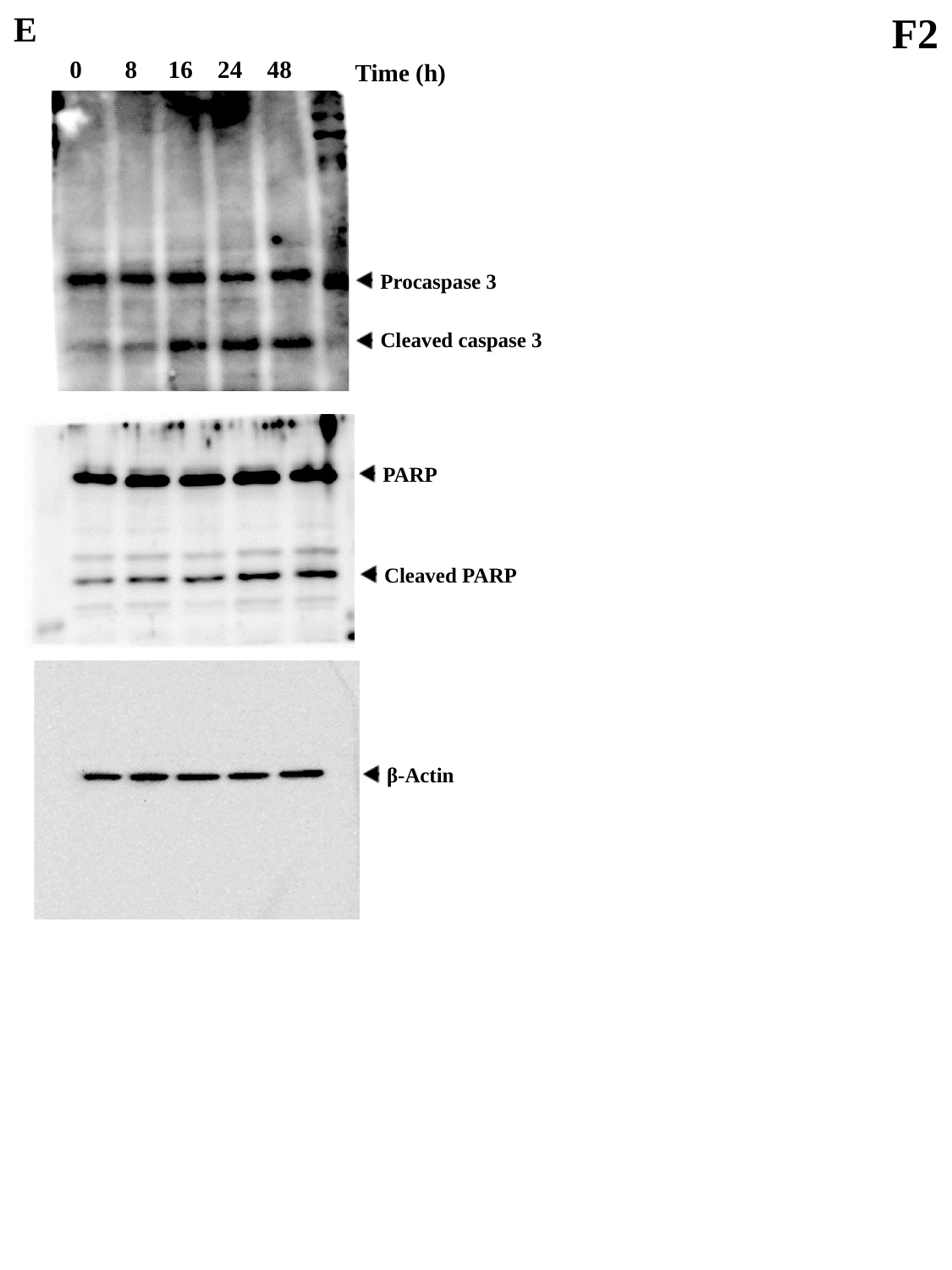

E
 F2
 0 8 16 24 48
Time (h)
Procaspase 3
Cleaved caspase 3
PARP
Cleaved PARP
β-Actin

## Slide 10
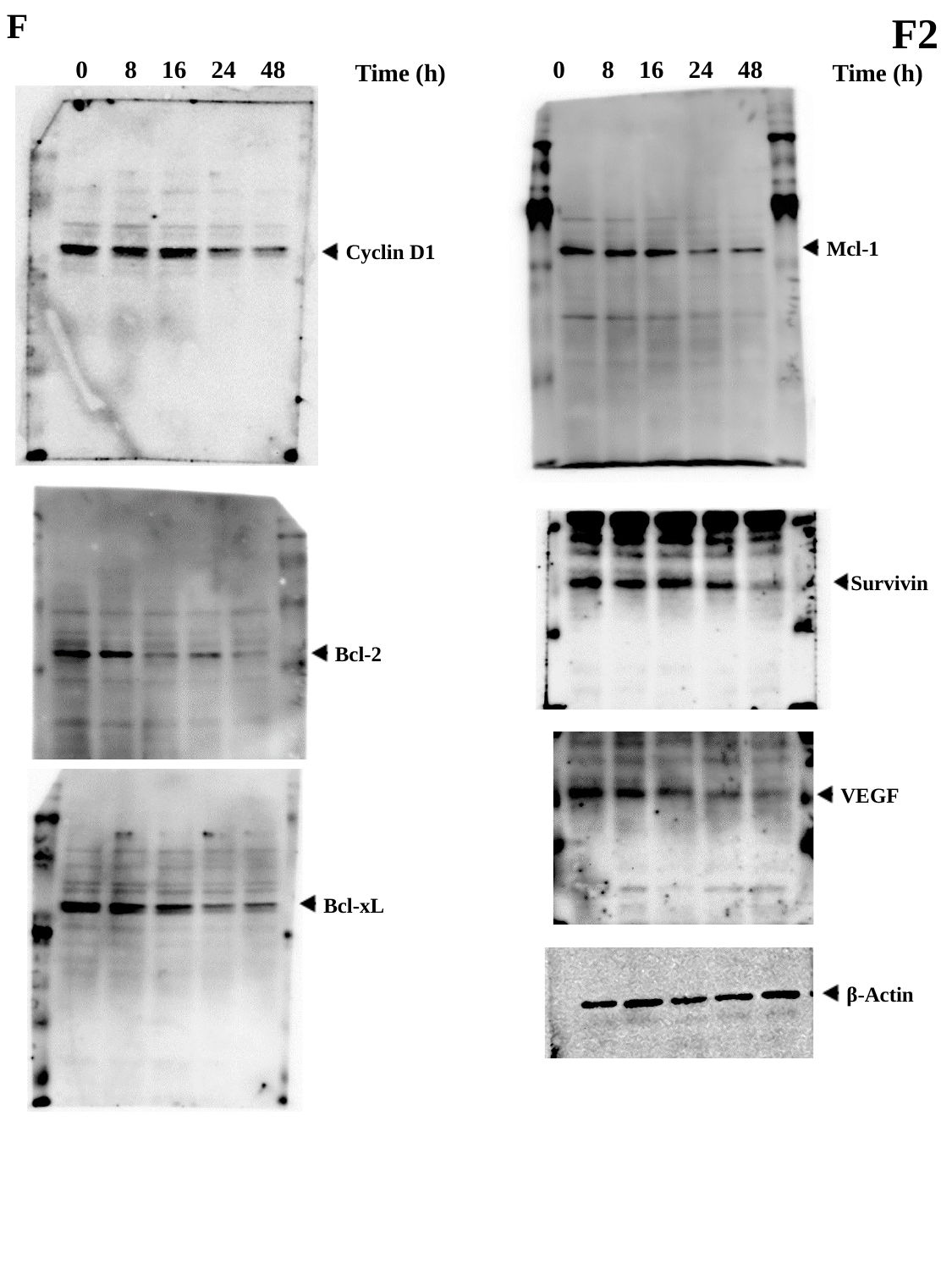

F2
F
 0 8 16 24 48
 0 8 16 24 48
Time (h)
Time (h)
Mcl-1
Cyclin D1
Survivin
Bcl-2
VEGF
Bcl-xL
β-Actin
